# Inducing multiple antibodies to treat squamous cell esophageal carcinoma

**DOI:** 10.1186/s12885-020-07466-0

**Published:** 2020-10-17

**Authors:** Isamu Hoshino, Yoshihiro Nabeya, Nobuhiro Takiguchi, Hisashi Gunji, Fumitaka Ishige, Yosuke Iwatate, Akiko Kuwajima, Fumiaki Shiratori, Rei Okada, Hideaki Shimada

**Affiliations:** 1 Division of Gastroenterological Surgery, Chiba Cancer Center, 666-2 Nitonacho, Chuo-ku, Chiba, 260-8717 Japan; 2 Department of Hepatobiliary and Pancreatic Surgery, Chiba Cancer Center, 666-2 Nitonacho, Chuo-ku, Chiba, Japan; 3 Medical & Biological Laboratories Co., Ltd, 4-5-3 Sakae, Naka-ku, Nagoya, 460-0008 Japan; 4 Department of Gastroenterological Surgery and Clinical Oncology, Graduate School of Medicine, Toho University, 6-11-1 Omori-Nishi, Ota-ku, Tokyo, 143-8541 Japan

**Keywords:** Autoantibody, Esophageal squamous cell carcinoma, TCGA

## Abstract

**Background:**

The positive response and the clinical usefulness of 14 serum antibodies in patients with esophageal squamous cell carcinoma (ESCC) were examined in this study. The Cancer Genome Atlas (TCGA) was used to investigate the frequency of gene expressions, mutations, and amplification of these 14 antigens and also the possible effects of antibody induction.

**Methods:**

Blood serum derived from 85 patients with ESCC was collected and analyzed for the 14 antibodies using ELISA. The prognosis between positive and negative antibodies were then compared. The antibody panel included LGALS1, HCA25a, HCC-22-5, and HSP70.

**Results:**

Patient serum was positive for all antibodies, except VEGF, with the positive rates ranging from 1.18 to 10.59%. Positive rates for LGALS1, HCA25a, HCC-22-5, and HSP70 were > 10%. TCGA data revealed that all antigen-related genes had little or no mutation or amplification, and hence an increase in gene expression affected antibody induction. The positive results from the panel accounted for the positive rate comparable to the combination of CEA and SCC. No significant association was observed between the presence of antibodies and disease prognosis.

**Conclusions:**

The detection rates of LGALS1, HCA25a, HCC-22-5, and HSP70 were 10% higher in patients with ESCC. Gene overexpression may be involved in such antibody production. These four antibodies were applied as a panel in comparison with conventional tumor markers. Moreover, it was confirmed that the combination of this panel and the conventional tumor markers significantly improved the positive rate.

## Background

Drugs that block immune checkpoints such as PD-1, PD-L1, and CTLA-4 have been developed in recent years [1, 2]. They have a significant effect on highly malignant cancers and are used effectively in cancer treatment. The principle underlying their mechanism of action is based on the appearance of cancer antigens that present cancer cell components on the basis of abnormal expression [1, 3]. T cells recognize the antigens present on the surface of cancer cells and begin attacking cancer cells [4]. The appearance of neoantigens and cancer antigens specific to cancer cells is believed to result from genetic mutations and gene expression abnormalities due to genetic instability [1, 5, 6]. Neoantigens and cancer antigens are recognized as foreign substances by the body, leading to a robust T-cell response. Recent research has reported about the possibility of correlating the effect of point inhibition therapy [7].

Futami et al. reported a new technology that quantitatively evaluates the level of immune response against cancer cells induced from a very small amount of blood [8]. They focused on the phenomenon wherein various cancer antigens and antibodies are increased in the blood when cancer immunotherapy is effective. However, neoantigens and cancer antigens are highly diverse (there are several hundred types), and there are large individual differences in terms of which antigens are expressed and which components are antigenic [9, 10]. Although neoantigens and cancer antigens are expected to predict the effects of treatment, the occurrence of antibodies against neoantigens is considered as a cancer patient-specific event.

We have extensively explored the usefulness of antibodies as biomarkers in our previous publications [11–14]. Compared with conventional tumor markers, it is believed that immune system markers such as antigen antibodies have the ability to recognize tumor cells during the early stages. Esophageal squamous cell carcinoma (ESCC) has a mutated p53, a tumor suppressor, at a frequency of around 80% [15–17]. Studies have reported that serum p53 antibody is expressed in approximately 20–30% of early-stage tumors [18, 19]. Moreover, NY-ESO-1 induces serum antibodies in the early stages of ESCC [20]. In addition to esophageal cancer, we had reported high expression rates of > 10% in lung cancer, liver cancer, stomach cancer, and prostate cancer [21].

In the present study, we evaluated the usefulness of antibodies extracted by serological identification, recombinant cDNA expression cloning (SEREX) method for treating ESCC, and also analyzed the frequency of gene mutations and abnormal expression using The Cancer Genome Atlas (TCGA, available online: https://cancergenome.nih.gov/) data [22].

## Methods

### Patients

Our study protocol was approved by the Institutional Review Board of Chiba Cancer Center (no. 21–26), and all patients provided their written informed consent. A total of 85 patients with ESCC who were treated in our hospital between October 2008 and August 2010 and 74 healthy controls were enrolled in this prospective study. The demographic characteristics of these patients are shown in Table 1. Healthy controls in the test cohort had no previous malignant disease. Survival follow-up was basically performed regularly at our hospital for patients treated at our hospital. Moreover, patients referred to other hospitals were followed up based on information provided by the transfer hospital or via telephone with approval. The observation period was from October 2008 to December 2018, with a median of 1120 days (20–3635 days).

**Table 1:** Patient details and clinicopathological features

	Esophageal cancer
**Number**	85
**Gender**	
Male	73 (85.9%)
Female	12 (14.1%)
**Mean age ± s.d. (years)**	68.2 ± 7.7
**Age range (years)**	45–85
**Depth of tumor invasion**	
T1	28 (32.9%)
T2	8 (9.4%)
T3	29 (34.1%)
T4	20 (23.5%)
**Lymph node metastasis**	
Positive	56 (65.9%)
Negative	29 (34.1%)
**Distant metastasis**	
Positive	19 (22.4%)
Negative	66 (77.6%)
**TNM stage**	
I	26 (30.6%)
II	7 (8.2%)
III	19 (22.4%)
IV	33 (38.8%)

### Purification of recombinant DNA

The SEREX method, which is an antigen screening method for ESCC, has been previously described [12]. For the expression and purification of recombinant protein, all antigen candidates were amplified by polymerase chain reaction. Additional processing was performed according to previously described protocols [23]. DNA sequencing analysis was performed to confirm that the correct sequence was inserted into the constructed plasmid.

### Detection of serum antibodies by ELISA

Serum samples obtained from patients and healthy controls were analyzed by ELISA, as previously described [24]. The signal of all antibodies was evaluated by calculating the difference in absorbency between plante wells containing antibodies and wells containing phosphate-buffered saline.

### Assay cut-off values

The cut-off values indicating positive reactivity were defined as optical density values greater than the mean value obtained from healthy controls + 7 S.D. for p90 antibody; + 6 S.D. for c-myc, KM-HN-1, and Sui1 antibodies; + 5 S.D. for p62, AnnexinII, cyclinB1, HSP70, and HSP40 antibodies; + 4 S.D. for LGALS1, HCA25a, and PRX6 antibodies; and + 3 S.D. for HCC-22-5 and VEGF antibodies [23, 25, 26]. The specificity of the assay was calculated as the percentage of healthy controls from whom negative results were obtained, and the specificity of all antibodies was 100% using these cut-off values.

### Frequency of gene mutation, gene expression, and survival analysis

In addition to antibody expression, cBioPortal (available online: https://www.cbioportal.org/) and UALCAN (available online: http://ualcan.path.uab.edu/) were used to investigate gene mutation and expression [27].. These web portals were also used to analyze survival rates based on gene expression levels. Survival analysis with *P* < 0.05 was considered to be statistically significant.

### Statistical analysis

All statistical analyses were performed using SPSS version 17.0 (Chicago, Ill., USA), Microsoft Excel (Redmond, Wash., USA), or GraphPad Prism software (La Jolla, Calif., USA). A chi-square test or a Fisher’s direct test was conducted to determine when the proportion of positive results differed significantly between cancer patients and healthy controls and to correlate individual and complex antibody assay positive results with clinical parameters. Identified. The correlation between overall survival and antibody status was calculated using the log rank test, and the results are presented as a curve using the Kaplan–Meier method. For all tests, *P* values < 0.05 (two-sided *t*-test) were considered to indicate statistical significance.

## Results

### Positive results of individual antibodies

**Fig. 1:**
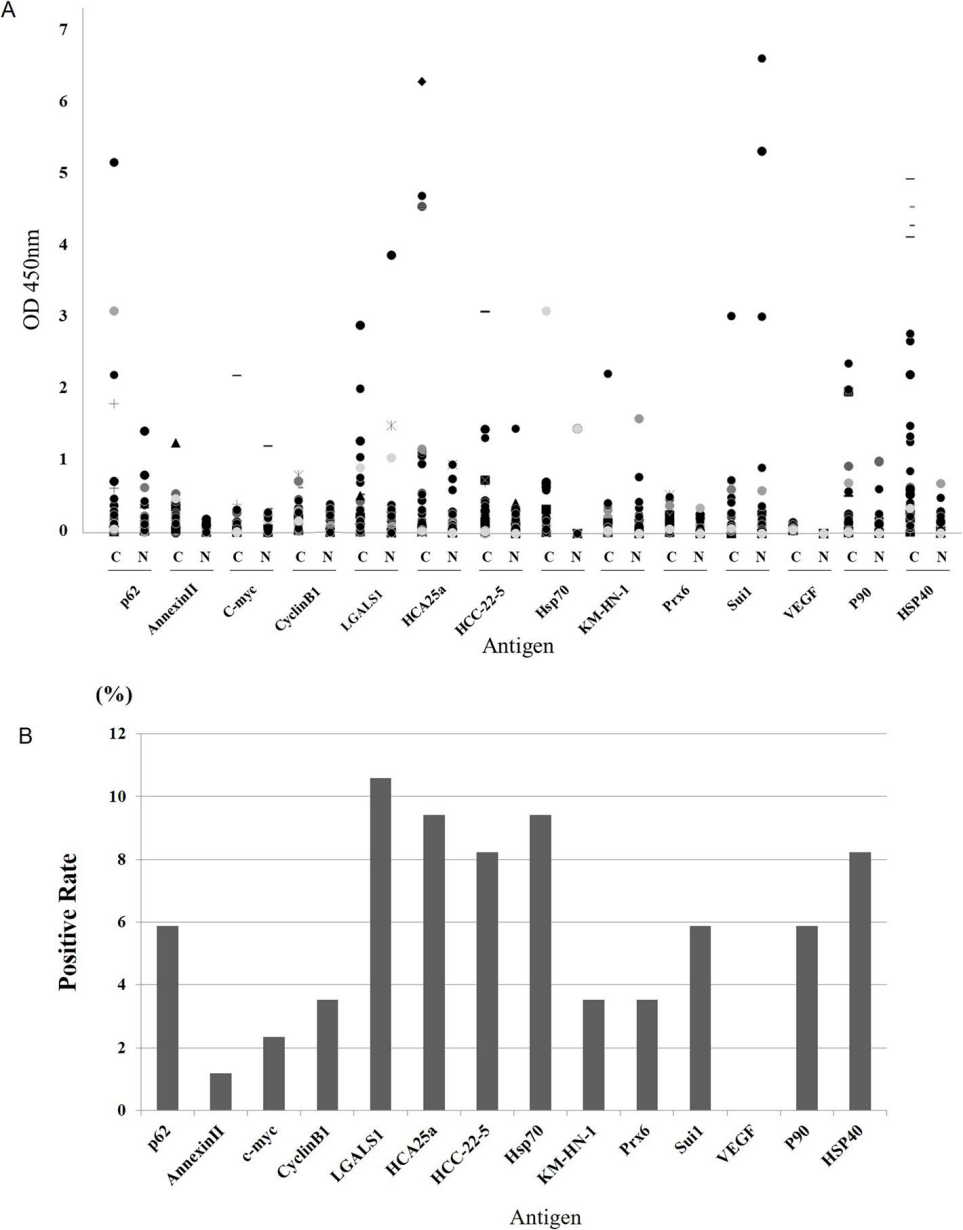
Enzyme-linked immunosorbent assay (ELISA) antibody titers of individual patients and normal controls for antibodies. **a** Scatter plots of optical density (OD) values of antibodies from ESCC sera and normal sera. **b** The sensitivity of each antibody is shown on the bar graph

The presence of autoantibodies is demonstrated for one concentration of antigen in the scatter plots in Fig. 1a. All antibodies, except VEGF, showed some positive result ranging from 1.18 to 10.59%. Among them, LGALS1, HCA25a, HCC-22-5, and HSP70 demonstrated a positive response of approximately 10%, which was higher than that of the other tested antibodies (Fig. 1b).

### Prognostic impact of antibodies and gene expression

**Fig. 2:**
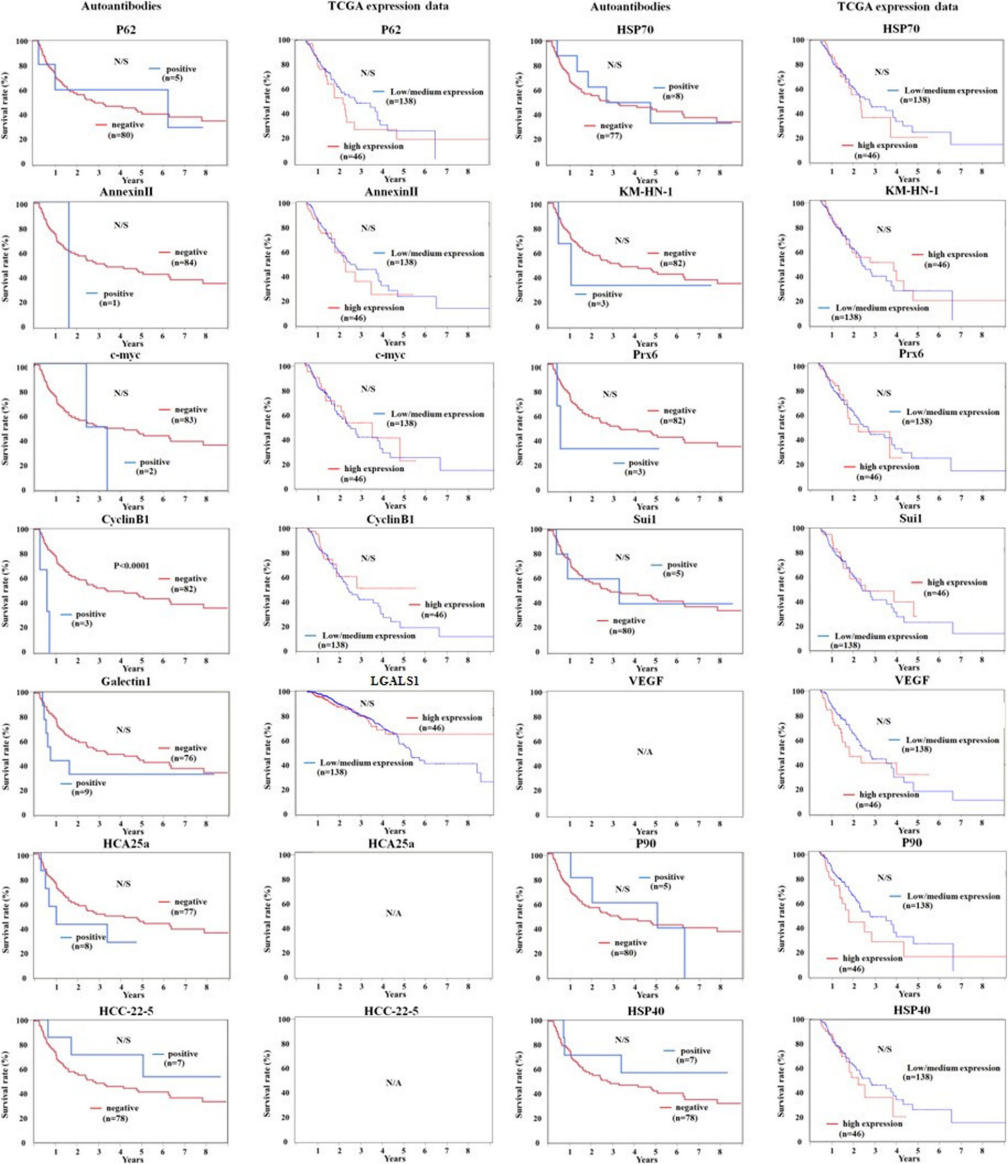
Prognostic role of antibodies or gene expression. The survival curve plotted are for a specific molecule based on the antibody level or the expression data of TCGA (UALCAN)

Prognosis based on the antibody status was examined for 14 antibodies, but there was no correlation between prognosis and positive or negative status of any of these 14 antibodies (Fig. 2). Furthermore, we compared the cancer prognosis with the level of gene expression using TCGA data but did not find any correlation (HCA25a and HCC-22-5 had no data) (Fig. 2).

### Relationship between antibody expression and gene expression, gene mutation, and gene amplification

The 14 antibodies examined in this study were analyzed using TCGA data for their gene expression levels, mutation rate, and frequency of gene amplification (Table 2). All antibodies, except KM-HN-1, exhibited a significant increase in gene expression in the cancer tissue compared to that in the normal tissue (HCA25a and HCC-22-5 had no data). However, the frequency of gene mutations was in the range of 0–1.85%, and no gene amplification was observed in any gene (Table 2).

**Table 2:** Relationship between antibody expression and gene expression, gene mutation, gene amplification

			Elisa	TCGA		
Gene Symbol	GenBank Accession number	Gene Description	Sensitivity (%)	Gene Expression	Mutation Frequency (%)	Gene Amplification (%)
p62	NM 003900	Phosphotyrosine-Independent Ligand For The Lck SH2 Domain Of 62 KDa	5.9	normal < tumor	0	0
Annexin AII	NM 004039	Annexin A2	1.2	normal < tumor	0	0
c-myc	NM 002467	MYC Proto-Oncogene, BHLH Transcription Factor	2.4	normal < tumor	0	0
Cyclin B1	NM 031966	Cyclin B1	3.5	normal < tumor	0	0
LGALS1	NM 001540	Heat Shock Protein Family B (Small) Member 1	10.6	normal < tumor	0	0
HCA25a	AF 469043	Hepatocellulara carcinoma-associated antigens, HCA25a	9.4	N/A	N/A	N/A
HCC-22-5	NM 004683	Hepatocellulara carcinoma-associated antigens, HCC-22-5	8.2	N/A	N/A	N/A
HSP70	NM 004134	Heat Shock Protein Family A (Hsp70) Member 4	9.4	normal < tumor	0	0
KM-HN-1	NM 152775	Coiled-Coil Domain Containing 110	3.5	normal > tumor	1.85	0
Prx6	NM 004905	Peroxiredoxin 6	3.5	normal < tumor	0	0
Sui1	NM 005801	Eukaryotic Translation Initiation Factor 1	5.9	normal < tumor	0	0
VEGF	AF 486837	Vascular Endothelial Growth Factor A	0.0	normal < tumor	0	0
p90	AF 334474	Transferrin Receptor	5.9	normal < tumor	0	0
HSP40	NM 006145	DnaJ Heat Shock Protein Family (Hsp40) Member B1	8.2	normal < tumor	0.93	0

Gene Expression: normal < tumor means that expression levels in tumor was signicicantly higher than the expression levels in normal tissue. Normal > tumor means that expression levels in tumor was signicicantly lower than the expression levels in normal tissue

### Antibody panel of LGALS1, HCA25a, HCC-22-5, and HSP70 for the diagnosis of ESCC

**Fig. 3:**
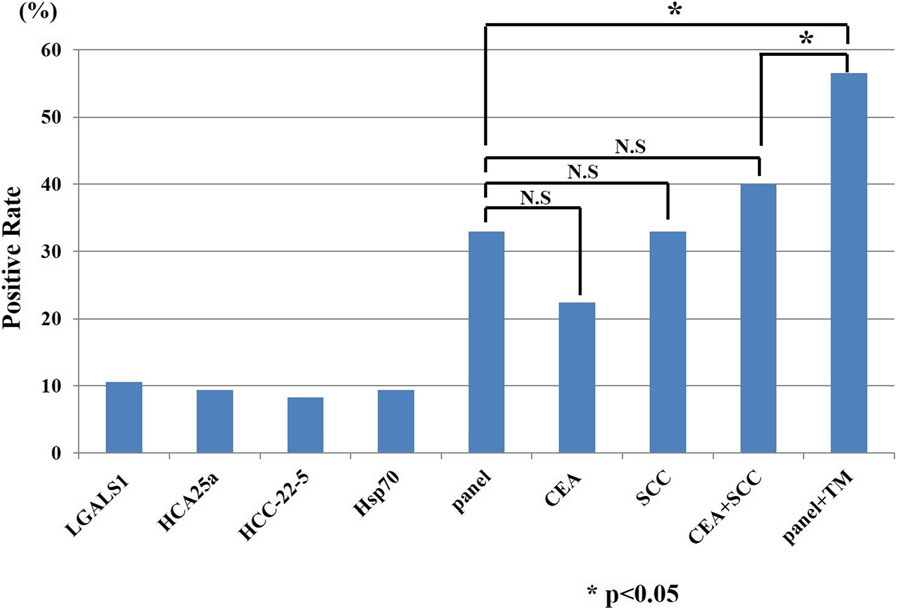
Sensitivity of antibodies with traditional tumor markers, CEA and SCC. TM means tumor markers (CEA + SCC)

The diagnostic abilities and the sensitivity of panels containing these four autoantibodies to detect ESCC was further investigated. First, we compared the sensitivity with that of the conventional tumor markers CEA and SCC. In the validation cohort, the sensitivity rates of CEA and SCC were 22.4 and 32.9%, respectively, and the sensitivity of the four-antibody panel was 32.9%. No significant difference was detected between the sensitivity of the panel and that of conventional tumor markers. There were also no significant differences between the combination and the panel (Fig. 3). The sensitivity rates of the four-antibody panel, CEA, and SCC were significantly higher than the sensitivity of the four autoantibody panel alone or the combination of conventional tumor markers. The positive status of the panel was not associated with any clinicopathological factors (Table 3). However, 7 of 24 cStageI cases were positive, suggesting the possibility of exhibiting panel-positive even in patients with early-stage ESCC.

**Table 3:** Patient details of panel positive in ESCC patients

	Panel		
**Positive**	**-**	**+**	
**Number**	58 (67.1%)	27 (32.9)	
**Gender**			
Male (%)	49 (57.6)	25 (29.4)	*p*=0.490
Female	9 (10.6)	2 (2.4)	
**Mean age ± s.d. (years)**	68.4 ± 7.5	70.5 ± 8.4	
**Age range (years)**	45-84	56-85	
**Depth of tumor invasion**			
T1	18 (21.2)	10 (11.8)	*p*=0.514
T2	6 (7.1)	2 (2.4)	
T3	23 (27.0)	6 (7.1)	
T4	11 (12.9)	9 (10.6)	
Lymph node metastasis			
Positive	38 (44.7)	18 (21.2)	*p*=0.887
Negative	20 (23.5)	9 (10.6)	
**Distant metastasis**			
Positive	14 (16.5)	5 (5.9)	p=0.765
Negative	44 (51.8)	22 (25.9)	
**TNM stage**			
I	17 (20.0)	7 (8.2)	*p*=0.999
II	7 (8.2)	4 (4.7)	
III	16 (18.8)	7 (8.2)	
IV	18 (21.2)	9 (10.6)	

### Prognostic role of antibody panel in patients with ESCC

**Fig. 4:**
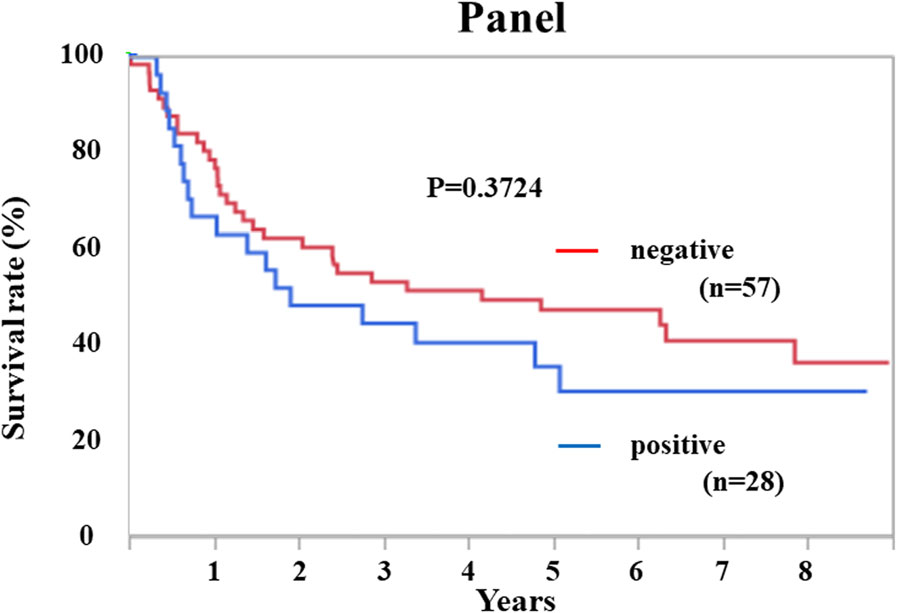
Overall survival curves with antibody positive groups and antibody negative groups

The survival rates of the antibody-panel-positive and -negative groups were compared (Fig. 4). Although the antibody-panel-positive group exhibited a relatively worse prognosis than the panel-negative group, there was no statistical difference between the two groups (*P* = 0.372).

## Discussion

In the present study, we examined patients with esophageal cancer for an increase in the expression levels of 14 autoantibodies and further evaluated the effects of gene expression, mutation, and amplification on the expression of these autoantibodies using TCGA data. All antibodies, except VEGF, demonstrated a certain positive expression rate compared with control. In addition, there was little or no mutation or amplification for any gene. We believe that an increase in gene expression might be responsible for the increased expression of these antibodies. Among the 14 autoantibodies, LGALS1, HCA25a, HCC-22-5, and HSP70 demonstrated a relatively high expression level compared to that of control, and hence they were extracted for further investigation. The expression levels of CEA and SCC-Ag, conventional tumor markers, were evaluated and compared with control. An increased expression level of the four antibodies was not inferior to the combination of CEA and SCC. Furthermore, the combination of the panel and the tumor marker significantly improved the positive rate compared with their own positive rates.

In our previous studies, we observed that serum p53 antibodies appeared at a higher rate in patients with ESCC [19, 28]. TCGA data confirmed that 156 (68.7%) of 227 patients with ESCC have p53 gene mutations. According to previous reports, the positive rate of p53-Ab is considered to be approximately 20% in patients with early-stage esophageal cancer and ≥ 30% in patients with advanced cancer [19, 29]. TCGA data demonstrated the presence of p53 gene mutation irrespective of early or advanced cancer, and it appears that gene mutation is significantly involved in the appearance of p53 antibodies. This is another reason that p53 antibodies are detected in patients with early-stage cancer.

It is known that when gene mutation occurs in p53, there is an increase in the expression of abnormal genes and abnormal proteins. When an antibody appears, it is considered to be the result of a series of flaws from a gene mutation accompanied by the appearance of an abnormal gene or abnormal protein. In contrast, NY-ESO-1 antibodies have been reported in several types of cancers [30]. Previous studies have reported positive expression rates of antibodies of 32.0, 12.3, 12.1, 10.5, 10.3, 8.4, and 7.1% for ESCC, lung cancer, liver cancer, prostate cancer, colon cancer, and breast cancer, respectively. However, according to TCGA data, the frequencies of gene mutation of each cancer type were 0, 0.24, 0.27, 0.9, 0.09, 0.32, and 0.09% and the gene amplification rates were 0, 1.5, 1.9, 2.7, 0.3, 0.65, and 0.8%, respectively. Even when both mutation rates are combined, the frequency ranges from 0% to several percentage. Gene expression did not increase in esophageal cancer and breast cancer, but increased expression was observed in other cancer types (*P* = 0.06 for esophageal cancer, with a tendency to increase). Sato et al. reported that gene expression levels were increased in ESCC and HCC [31]. However, because the frequency of gene mutation and gene amplification is not high, a mechanism not associated with p53 may be involved in these gene expression increases. In malignant mesothelioma, the expression of NY-ESO-1 is increased through histone deacetylase inhibitor [32]. Studies conducted using ovarian cancer cell lines have reported that the expression level of NY-ESO-1 was increased using DNA methylation inhibitors [33], indicating that epigenetic mechanisms such as acetylation and methylation may be involved in NY-ESO-1 expression. We found almost no gene mutation or amplification for the 14 antigens and antibodies that we selected in our study. Except for two antigens for which data could not be confirmed, and except for KM-HN-1, there was a significant increase in gene expression in the cancer tissue compared to that in the normal tissue. It is possible that mechanisms other than gene mutation and amplification were involved in increasing the expression and appearance of antigens.

We had previously reported that using antibody panels in gastric cancer can provide results that surpass the results provided by existing tumor markers [23]. In the present study, we extracted four antibodies that are considered to have a relatively high positive rate among the 14 antibodies and evaluated them as a panel. We found that the positive rate intensely improved to 32.9%. Moreover, it was confirmed that the combination of CEA and SCC, conventional tumor markers, significantly increased the sensitivity compared with the use of the panel alone or the combination of tumor markers. Antibodies, which are considered to have an insufficient positive rate by themselves, may improve their usefulness by being added to a panel or when used in combination with conventional tumor markers. The specificity of any antibody was 100% at the cut-off value used in the present study in the current cohort, and their usefulness is regarded as appropriate considering that the specificity of existing tumor markers is not sufficiently high [34]. In recent years, microsatellite instability, tumor mutational burden, and other genetic mutations have received attention as biomarkers for their effects of immune checkpoint inhibitors [35–37]. This is because they are believed to be involved in increasing the appearance of targeted neoantigens and cancer antigens. There is a possibility that the increase in antibodies we examined in the present study could correlate with an effect of immune checkpoint inhibitors; however, this assumption needs to be clarified in future clinical trials.

A limitation of the present study was that it was conducted at a single facility. However, the ongoing clinical trials in esophageal cancer and hepatocellular carcinoma, including studies on the antibodies we have examined in the present study, are being conducted in collaboration with other institutions. We anticipate that the usefulness of these antibodies will become clearer in the near future. Since data such as gene expression, mutation, and amplification were analyzed using TCGA public data, it is not the case used for autoantibody analysis in this study, and a detailed evaluation of differences among races and others was not possible.

## Conclusions

This study evaluated the positive rate of antibodies in patients with esophageal cancer. Moreover, the mechanism of antibody expression was examined using TCGA data in terms of gene expression, mutation, and amplification. We believe that this study suggests the usefulness of biomarker increases using multiple antibodies as a panel.

## Data Availability

All analyzed data are included in this published article. The original data are available upon request to the corresponding author.

## Abbreviations

CEA: Carcinoembryonic antigen
c-myc: MYC proto-oncogene, BHLH transcription factor
ELISA: Enzyme-linked immunosorbent assay
ESCC: Esophageal squamous cell carcinoma
HCA25a: Hepatocellular carcinoma-associated antigen, HCA25a
HCC-22-5: Hepatocellular carcinoma-associated antigen, HCC-22-5
HSP40: DnaJ heat shock protein family (Hsp40) member B1
HSP70: Heat shock protein family a (Hsp70) member 4
Prx6: Peroxiredoxin 6
p62: Phosphotyrosine-independent ligand for the lck SH2 domain Of 62 KDa
SEREX: Serological analysis of recombinant tumor cDNA expression libraries
SCC: Squamous cell carcinoma antigen
TCGA: The cancer genome atlas
VEGF: Vascular endothelial growth factor a

## References

[1] Darvin P, Toor SM, Sasidharan Nair V, Elkord E (2018). Immune checkpoint inhibitors: recent progress and potential biomarkers. Exp Mol Med.

[2] Fan Y, Zhang C, Jin S, Gao Z, Cao J, Wang A (2019). Progress of immune checkpoint therapy in the clinic (review). Oncol Rep [review].

[3] Vigneron N (2015). Human tumor antigens and cancer immunotherapy. Biomed Res Int.

[4] Baumeister SH, Freeman GJ, Dranoff G, Sharpe AH (2016). Coinhibitory pathways in immunotherapy for cancer. Annu Rev Immunol.

[5] Chae YK, Anker JF, Oh MS, Bais P, Namburi S, Agte S (2019). Mutations in DNA repair genes are associated with increased neoantigen burden and a distinct immunophenotype in lung squamous cell carcinoma. Sci Rep.

[6] Nouri Rouzbahani F, Shirkhoda M, Memari F, Dana H, Mahmoodi Chalbatani G, Mahmoodzadeh H (2018). Immunotherapy a new Hope for cancer treatment: a review. Pak J Biol Sci.

[7] Yi M, Qin S, Zhao W, Yu S, Chu Q, Wu K (2018). The role of neoantigen in immune checkpoint blockade therapy. Exp Hematol Oncol.

[8] Futami J, Nonomura H, Kido M, Niidoi N, Fujieda N, Hosoi A (2015). Sensitive multiplexed quantitative analysis of autoantibodies to cancer antigens with chemically S-Cationized full-length and water-soluble denatured proteins. Bioconjug Chem.

[9] Pan RY, Chung WH, Chu MT, Chen SJ, Chen HC, Zheng L (2018). Recent development and clinical application of cancer vaccine: targeting neoantigens. J Immunol Res.

[10] Hutchison S, Pritchard AL (2018). Identifying neoantigens for use in immunotherapy. Mamm Genome.

[11] Shimada H, Nakashima K, Ochiai T, Nabeya Y, Takiguchi M, Nomura F (2005). Serological identification of tumor antigens of esophageal squamous cell carcinoma. Int J Oncol.

[12] Nakashima K, Shimada H, Ochiai T, Kuboshima M, Kuroiwa N, Okazumi S (2004). Serological identification of TROP2 by recombinant cDNA expression cloning using sera of patients with esophageal squamous cell carcinoma. Int J Cancer.

[13] Kuboshima M, Shimada H, Liu TL, Nakashima K, Nomura F, Takiguchi M (2006). Identification of a novel SEREX antigen, SLC2A1/GLUT1, in esophageal squamous cell carcinoma. Int J Oncol.

[14] Hoshino I, Nabeya Y, Takiguchi N, Gunji H, Ishige F, Iwatate Y (2020). Prognostic impact of p53 and/or NY-ESO-1 autoantibody induction in patients with gastroenterological cancers. Ann Gastroenterol Surg..

[15] Lin DC, Hao JJ, Nagata Y, Xu L, Shang L, Meng X (2014). Genomic and molecular characterization of esophageal squamous cell carcinoma. Nat Genet.

[16] Gao YB, Chen ZL, Li JG, Hu XD, Shi XJ, Sun ZM (2014). Genetic landscape of esophageal squamous cell carcinoma. Nat Genet.

[17] Zhang L, Zhou Y, Cheng C, Cui H, Cheng L, Kong P (2015). Genomic analyses reveal mutational signatures and frequently altered genes in esophageal squamous cell carcinoma. Am J Hum Genet.

[18] Shimada H, Takeda A, Arima M, Okazumi S, Matsubara H, Nabeya Y (2000). Serum p53 antibody is a useful tumor marker in superficial esophageal squamous cell carcinoma. Cancer.

[19] Shimada H, Nabeya Y, Okazumi S, Matsubara H, Funami Y, Shiratori T (2002). Prognostic significance of serum p53 antibody in patients with esophageal squamous cell carcinoma. Surgery.

[20] Xu YW, Peng YH, Chen B, Wu ZY, Wu JY, Shen JH (2014). Autoantibodies as potential biomarkers for the early detection of esophageal squamous cell carcinoma. Am J Gastroenterol.

[21] Matejcic M, Gunter MJ, Ferrari P (2017). Alcohol metabolism and oesophageal cancer: a systematic review of the evidence. Carcinogenesis.

[22] Nagy P, Johansson S, Molloy-Bland M (2016). Systematic review of time trends in the prevalence of helicobacter pylori infection in China and the USA. Gut Pathog.

[23] Hoshino I, Nagata M, Takiguchi N, Nabeya Y, Ikeda A, Yokoi S (2017). Panel of autoantibodies against multiple tumor-associated antigens for detecting gastric cancer. Cancer Sci.

[24] Shimada H, Fau KA, Shiratori T, Fau ST, Nomura F, Nomura F (2009). Detection of anti-CUEC-23 antibodies in serum of patients with esophageal squamous cell carcinoma: a possible new serum marker for esophageal cancer. (0944-1174 (print)). J Gastroenterol.

[25] Zhang JY, Casiano CA, Peng XX, Koziol JA, Chan EK, Tan EM (2003). Enhancement of antibody detection in cancer using panel of recombinant tumor-associated antigens. Cancer Epidemiol Biomark Prev.

[26] Ushigome M, Nabeya Y, Soda H, Takiguchi N, Kuwajima A, Tagawa M (2018). Multi-panel assay of serum autoantibodies in colorectal cancer. Int J Clin Oncol.

[27] Chandrashekar DS, Bashel B, Balasubramanya SAH, Creighton CJ, Ponce-Rodriguez I, Chakravarthi BVSK (2017). UALCAN: a portal for facilitating tumor subgroup gene expression and survival analyses. Neoplasia.

[28] Suzuki T, Yajima S, Ishioka N, Nanami T, Oshima Y, Washizawa N (2018). Prognostic significance of high serum p53 antibody titers in patients with esophageal squamous cell carcinoma. Esophagus.

[29] Shimada H (2018). p53 molecular approach to diagnosis and treatment of esophageal squamous cell carcinoma. Ann Gastroenterological Surg.

[30] Oshima Y, Shimada H, Yajima S, Nanami T, Matsushita K, Nomura F (2016). NY-ESO-1 autoantibody as a tumor-specific biomarker for esophageal cancer: screening in 1969 patients with various cancers. J Gastroenterol.

[31] Sato S, Noguchi Y, Wada H, Fujita S, Nakamura S, Tanaka R (2005). Quantitative real-time RT-PCR analysis of NY-ESO-1 and LAGE-1a mRNA expression in normal tissues and tumors, and correlation of the protein expression with the mRNA copy number. Int J Oncol.

[32] Bensaid D, Blondy T, Deshayes S, Dehame V, Bertrand P, Grégoire M (2018). Assessment of new HDAC inhibitors for immunotherapy of malignant pleural mesothelioma. Clin Epigenet.

[33] Srivastava P, Paluch BE, Matsuzaki J, James SR, Collamat-Lai G, Taverna P (2015). Immunomodulatory action of the DNA methyltransferase inhibitor SGI-110 in epithelial ovarian cancer cells and xenografts. Epigenetics..

[34] Takeshita N, Hoshino I, Mori M, Akutsu Y, Hanari N, Yoneyama Y (2013). Serum microRNA expression profile: miR-1246 as a novel diagnostic and prognostic biomarker for oesophageal squamous cell carcinoma. Br J Cancer.

[35] Carbone DP, Reck M, Paz-Ares L, Creelan B, Horn L, Steins M (2017). First-line nivolumab in stage IV or recurrent non-small-cell lung cancer. N Engl J Med.

[36] Rizvi H, Sanchez-Vega F, La K, Chatila W, Jonsson P, Halpenny D (2018). Molecular determinants of response to anti-programmed cell death (PD)-1 and anti-programmed death-ligand 1 (PD-L1) blockade in patients with non-small-cell lung cancer profiled with targeted next-generation sequencing. J Clin Oncol.

[37] Samstein RM, Lee CH, Shoushtari AN, Hellmann MD, Shen R, Janjigian YY (2019). Tumor mutational load predicts survival after immunotherapy across multiple cancer types. Nat Genet.

